# Investigation of False Positive Results with an Oral Fluid Rapid HIV-1/2 Antibody Test

**DOI:** 10.1371/journal.pone.0000185

**Published:** 2007-01-31

**Authors:** Krishna Jafa, Pragna Patel, Duncan A. MacKellar, Patrick S. Sullivan, Kevin P. Delaney, Tracy L. Sides, Alexandra P. Newman, Sindy M. Paul, Evan M. Cadoff, Eugene G. Martin, Patrick A. Keenan, Bernard M. Branson, for the OraQuick Study Group

**Affiliations:** 1 Division of HIV/AIDS Prevention, National Center for HIV, STD and TB Prevention, Centers for Disease Control and Prevention, Atlanta, Georgia, United States of America; 2 Infectious Disease Epidemiology, Prevention and Control Division, Minnesota Department of Health, Saint Paul, Minnesota, United States of America; 3 Wisconsin Division of Public Health, Madison, Wisconsin, United States of America; 4 Epidemiology Program Office, Office of Workforce and Career Development, Centers for Disease Control and Prevention, Atlanta, Georgia, United States of America; 5 New Jersey Department of Health and Senior Services, Division of HIV/AIDS Services, Trenton, New Jersey, United States of America; 6 Department of Pathology and Laboratory Medicine, Robert Wood Johnson Medical School, University of Medicine and Dentistry of New Jersey, New Brunswick, New Jersey, United States of America; 7 Department of Family Medicine and Community Health, University of Minnesota School of Medicine, Minneapolis, Minnesota, United States of America; University of California, San Francisco, United States of America

## Abstract

**Background:**

In March 2004, the OraQuick® rapid HIV antibody test became the first rapid HIV test approved by the US Food and Drug Administration for use on oral fluid specimens. Test results are available in 20 minutes, and the oral fluid test is non-invasive. From August 2004–June 2005, we investigated a sudden increase in false-positive results occurring in a performance study of OraQuick® oral-fluid rapid HIV tests in Minnesota.

**Methodology/Principal Findings:**

In a field investigation, we reviewed performance study data on oral-fluid and whole-blood OraQuick® rapid HIV test device lots and expiration dates and assessed test performance and interpretation with oral-fluid and whole-blood specimens by operators who reported false-positive results. We used multivariate logistic regression to evaluate client demographic and risk characteristics associated with false-positive results. Next, we conducted an incidence study of false-positive OraQuick rapid HIV tests in nine US cities and tested both oral-fluid and finger-stick whole-blood specimens from clients; reactive tests were confirmed with Western blot. Sixteen (4.1%) false-positive oral-fluid results occurred in the performance study from April 15, 2004 through August 31, 2004 with unexpired devices from six test lots among 388 HIV-uninfected clients (specificity, 95.9%; 95% CI: 93.4–97.6). Three test operators who had reported false-positive results performed and interpreted the test according to package-insert instructions. In multivariate analysis, only older age was significantly associated with false-positive results (adjusted odds ratio = 4.5, 95% CI: 1.2–25.7). In the incidence study, all valid oral-fluid and whole-blood results from 2,268 clients were concordant and no false-positive results occurred (100% specificity).

**Conclusions/Significance:**

The field investigation did not identify a cause for the increase in false-positive oral-fluid results, and the incidence study detected no false-positive results. The findings suggest this was an isolated cluster; the test's overall performance was as specified by the manufacturer.

## Introduction

In March 2004, the OraQuick® rapid HIV-1 antibody test (Orasure Technologies, Bethlehem, Pennsylvania, USA) became the first rapid Human Immunodeficiency Virus (HIV) test approved by the US Food and Drug Administration (FDA) for use on oral fluid specimens. In June 2004, the FDA approved the test for HIV-2 antibody detection in oral fluid and a name change to OraQuick® Advance Rapid HIV-1/2 Antibody Test [1]. OraQuick^1^ is a qualitative, visually read lateral-flow immunoassay intended for point-of-care use and is waived for use on oral fluid, finger-stick, and venipuncture whole blood specimens under CLIA (the Clinical Laboratory Improvement Amendments of 1988) [2]. The test is performed on an oral fluid specimen, collected by swabbing the flat pad of the test device once around the outer surface of the upper and lower gums, or on 5 µL of whole blood [1]. A reactive test develops reddish-purple lines in both the test and control zones of the device, while a non-reactive test develops a reddish-purple line in the control zone of the device and no line in the test zone [1]. A test is considered invalid if the control or test line develops outside either the control or test zone, if no control line develops, or if a red background in the result window makes it difficult to read the result after 20 minutes [1]. Results are read in 20–40 minutes [1]. Data submitted by the manufacturer as part of an FDA review for approval indicated a specificity of 99.8% (95% confidence interval [CI]: 99.6–99.9) on oral fluid and 100% (95% CI: 99.7–100) on whole blood [1].

As part of its Advancing HIV Prevention initiative, the Centers for Disease Control and Prevention (CDC) supports rapid HIV testing methods to increase HIV testing and awareness of HIV status [3]. In July 2002, the University of Minnesota's Department of Family Practice initiated a CDC-sponsored study to evaluate the performance of OraQuick to test persons with unknown HIV status in outreach settings [4]. In July 2004, the Minnesota Department of Health requested CDC to investigate a sudden increase in false-positive results with the oral-fluid rapid HIV tests. Test operators described the false-positives as having qualitatively different test lines, but test-line intensity and color were not recorded [4]. In this paper we report on: (1) a field investigation to evaluate false-positive device characteristics, to assess test operator practices, and to retrospectively evaluate performance study client characteristics associated with false-positive test results; and (2) an incidence study of false-positive OraQuick test results to prospectively evaluate client characteristics associated with false-positive test results and device characteristics including test-line intensity and color.

## Methods

### Field Investigation

#### Definitions

A false-positive rapid HIV test result was defined as a reactive oral-fluid OraQuick test result followed by a negative FDA-approved serum or oral-fluid Western blot.

#### Device investigation

We reviewed performance study data for two time periods: July 1, 2002–April 14, 2004 and April 15, 2004–August 31, 2004. The latter corresponded to the time period during which 16 false-positive oral-fluid results were reported. To assess device characteristics associated with false-positive tests, we reviewed test storage conditions at the performance study site and examined expiration dates and manufacturer lot numbers of the tests yielding false-positive results. We requested the manufacturer to report any manufacturing discrepancies for implicated lots.

#### Operator investigation

We interviewed performance study operators, all of whom had performed and interpreted tests yielding false-positive results, about any unusual device characteristics for those tests. To assess operator practices while performing and interpreting the test we observed operators who reported ≥1 false-positive result as they prepared OraQuick devices, performed one oral-fluid and one finger-stick whole-blood test each on volunteer specimens, inserted devices into the developer solution, timed the test, and interpreted results.

#### Epidemiologic investigation

We constructed an epidemic curve of false-positive results that occurred during the performance study. To evaluate demographic and risk characteristics potentially associated with false-positive results, we examined client data collected at the time of testing during two time periods: July 1, 2002–April 14, 2004 and April 15, 2004–August 31, 2004. In univariate analyses, we used exact Mantel-Haenszel chi-square tests and exact 95% confidence limits for odds ratios. We included variables associated with false-positive results at *P*≤0.25 in univariate analysis in a multivariate logistic regression model. We performed all analyses with SAS (version 9.1, SAS Institute, Cary, North Carolina, USA).

### Incidence Study

#### Rationale

From February 1, 2005 through May 31, 2005, we conducted a multi-site study of the incidence of false positive results from oral-fluid OraQuick tests because (1) the false-positive results occurred at the end of the Minnesota performance study and it was unknown whether similar incidence would continue; (2) the initial field investigation was retrospective and information on host factors (e.g., pre-existing medical conditions and interfering substances) that might be associated with false-positive results could not be elicited; and, (3) the performance study did not record test line intensity and color, thereby limiting our ability to accurately describe false-positive results read within the 20–40 minute reading window and to determine the degree of under-ascertainment of faintly reactive devices among operators at other sites who had not reported any false positives. If a sufficient number of false-positive results occurred during the incidence study, we planned a case-control study to assess the associations of host factors with false-positive results.

#### Definitions

For the purpose of the incidence study, we defined a negative HIV test result as one in which the client's whole-blood rapid HIV test result was non-reactive. We defined a positive HIV test result as one with a reactive whole-blood rapid test followed by an FDA-approved positive serum Western blot.

#### Sample size

We based the sample size calculation for the incidence study on the FDA requirement that, in clinical trials of rapid HIV tests, the lower bound of the 95% confidence interval (CI) for specificity must be at least 98% [5]. With a false-positive incidence of 1.5%, a sample size of 1800 would yield a 95% CI for specificity of 97.9–99.0 [6]. If we observed a false-positive incidence ≥1.5%, the test would not meet FDA specificity requirements and there would be sufficient false-positives for us to proceed with the case-control study.

#### Study settings and methods

Three state health departments conducted the incidence study from February 1, 2005 through May 31, 2005 at HIV testing sites already using rapid tests in nine cities: Minneapolis, Minnesota; Madison and Milwaukee, Wisconsin; and Atlantic City, East Orange, Hackensack, Newark, New Brunswick and Trenton, New Jersey. Minnesota was invited to participate because the sudden increase in false-positives was reported in Minneapolis; other states volunteered to participate. Clients seeking HIV testing were eligible for enrollment into the incidence study if they were ≥18 years old and had no history of antiretroviral therapy, HIV vaccination, or a previous HIV diagnosis.

All incidence study participants consented to and received both whole-blood and oral-fluid OraQuick tests. Participants with concordant non-reactive rapid test results received a negative result, while those with concordant reactive rapid test results received a preliminary positive result. Participants with discordant rapid test results were informed of both results, offered confirmatory testing, and invited to participate in the case-control study. To assess rapid test specificity, any reactive rapid test result was followed by a ‘gold standard’ Genetic Systems HIV-1 Western blot (Bio-Rad Laboratories, Inc., Hercules, California, USA) on serum specimens. Since sensitivity was not under investigation in the incidence study, no additional testing was conducted on concordant non-reactive whole-blood and oral-fluid OraQuick tests (such additional testing was conducted in the performance study and is reported elsewhere [4]). All study operators had received previous training on administration and interpretation of OraQuick. To ensure identification of all false-positive results, we re-trained operators to interpret and record the intensity and color of all test lines observed.

#### Data collection and analysis

We used QDS™ Questionnaire Development System Version 2.3 (Nova Research Company, Bethesda, Maryland, USA) to record rapid-test results, color and intensity of test and control lines, invalid results, manufacturer lot number, and rapid-test operator initials and read times. Sites also reported Western blot results and routinely collected demographic and risk characteristics of testing clients enrolled as study participants. We calculated specificity with a standard formula and performed all analyses with SAS version 9.1.

#### Human subjects

As a public-health response to an increase in false-positive test results from an FDA-approved device, CDC determined the study did not require Institutional Review Board (IRB) review under human-subjects-protection guidelines. The Minnesota and Wisconsin IRBs of record concurred. To comply with human-subjects guidelines in New Jersey, the protocol was approved by the New Jersey Department of Health & Senior Services IRB of record.

## Results

### Field Investigation

#### Device investigation

**Figure 1:**
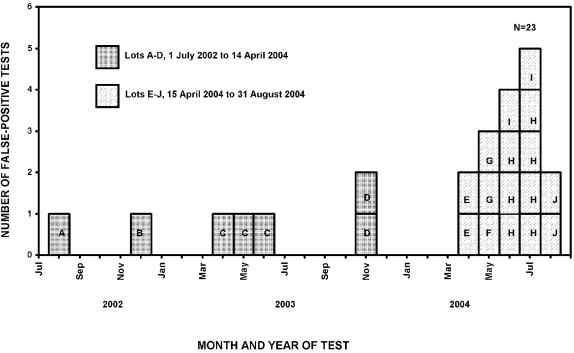
Epidemic curve of false-positive OraQuick rapid HIV antibody test results, oral fluid, by Month and Lot, University of Minnesota study, July 2002 through August 2004.

From July 1, 2002 through April 14, 2004 seven (0.3%) false-positive oral-fluid OraQuick results occurred with test devices from four different lots among 2017 HIV-uninfected clients (Fig. 1). From April 15, 2004 through August 31, 2004, 16 (4.1%) false-positive oral-fluid OraQuick results occurred with test devices from six different lots among 388 HIV-uninfected clients (specificity, 95.9%; 95% CI: 93.4–97.6) (Fig. 1). All false-positive tests were stored and used within the manufacturer's temperature specifications, and used before their expiration date. The manufacturer reported that all implicated lots were manufactured and shipped according to standard procedures and that all device components were within manufacturer specifications.

#### Operator investigation

Three of the four operators who had observed the 16 false-positive test results were interviewed; one had left the study and could not be contacted. All were involved in the performance study from start to finish and reported that most test lines they observed in devices with false-positive oral-fluid results were faint; some test lines were described as gray. When test lines were faint operators, who worked in teams conducting outreach testing, reported that they occasionally sought the opinion of another operator on whether the test was reactive or non-reactive. Operators did not report any unusual environmental conditions during the period when the increased number of false-positive tests was reported. We observed that all operators used the rapid test on volunteer oral-fluid and whole-blood specimens in accordance with the package insert and interpreted results correctly.

#### Epidemiologic investigation

No client demographic or risk factors were associated with false positive oral-fluid test results during July 1, 2002–April 14, 2004. Further host-factor analyses were restricted to 382 (94%) clients tested in the performance study during April 15, 2004–August 31, 2004 for whom complete demographic and risk data were available. In univariate analyses age, non-Black race, injection drug use, and male-to-male sex were associated with false-positive results at a threshold of *P*≤0.25 (Table 1). In the multivariate logistic regression model, only older age was significantly associated (*P* = 0.02) with false-positive oral-fluid results (Table 1).

**Table 1:** Demographic and risk characteristics of 382 clients who had a true-negative or false-positive oral fluid OraQuick Rapid HIV-1 Antibody Test result—University of Minnesota performance study, April 15, 2004 through August 31, 2004.

Client characteristics	Test Result				OR (95% CI)^a^	AOR (95% CI)
	True negative		False positive			
	n = 366	(%)	N = 16	(%)		
**Demographic Characteristics**						
**Age** b						
<37 years	173	(47)	3	(19)	1.0	1.0
≥37 years	193	(53)	13	(81)	**3.9 (1.0–21.5)**	**4.5 (1.2–25.7)** *
**Gender**						
Female	131	(36)	4	(25)	1.0	–
Male	235	(64)	12	(75)	1.7 (0.5–7.2)	–
**Race**						
Black	244	(67)	8	(50)	1.0	1.0
White	98	(27)	7	(44)	**2.2 (0.7–7.1)**	1.6 (0.4–6.4)
A/PI/AI/AN/Other	24	(6)	1	(6)	**1.3 (0.03–10.2)**	1.4 (0.03–12.2)
**Ethnicity**						
Hispanic	16	(4)	1	(6)	1.0	–
Non-Hispanic	350	(96)	15	(94)	1.5 (0.03–10.7)	–
**Risk Behaviors** c						
IDU	17	(5)	3	(19)	**4.7 (0.8–19.6)**	4.4 (0.5–28.5)
Sex partner of IDU	25	(7)	3	(19)	**3.1 (0.5–12.5)**	1.2 (0.2–7.0)
MSM or sex partner of MSM	11	(3)	2	(13)	**4.6 (0.5–24.2)**	4.1 (0.3–28.2)
Sex partner has HIV/AIDS	7	(2)	1	(6)	3.4 (0.1–29.4)	–
STD diagnosis	34	(9)	1	(6)	0.7 (0.02–4.5)	–
Sex for money	37	(10)	2	(13)	1.3 (0.1–5.9)	–
≥4 sex partners	106	(29)	4	(25)	0.8 (0.2–2.8)	–
No reported risk	201	(55)	7	(44)	0.6 (0.2–2.0)	–

OR, odds ratio; AOR, adjusted odds ratio; CI, confidence interval; A/PI/AI/AN/Other, Asian/Pacific Islander/American Indian/Alaska Native/Other; IDU, injection drug users; MSM, men who have sex with men; STD, sexually transmitted diseases.

a Values in bold font represent P≤0.25; variables included in the logistic regression model (see methods).

b Age was categorized into a dichotomous variable with 37 years as the cutoff as this was the median age of performance study clients during both July 1, 2002–April 14, 2004 and April 15, 2004–August 31, 2004.

c Risk behaviors are not mutually exclusive.

* *P*<0.05

### Incidence Study

#### Participant characteristics

Of 3067 eligible clients at all study sites who received a whole-blood rapid test, 2283 (74%) consented to an oral-fluid rapid test. Of 2283 clients who provided consent, 15 (0.6%) were excluded from analyses because either their test results were read before 20 minutes (n = 9) or their records had missing data (n = 6). Demographic and risk characteristics of the remaining 2268 clients are summarized in Table 2.

**Table 2:** Demographic characteristics and HIV risk behaviors of 2268 clients whose finger-stick whole blood and oral fluid specimens were tested with the OraQuick Advance rapid HIV test, by state—incidence study, February 2005 through May 2005.

Client characteristics	Minnesota		New Jersey		Wisconsin		All Sites	
	n = 1039	(%)	n = 304	(%)	n = 925	(%)	n = 2268	(%)
**Demographic Characteristics**								
**Age in years**								
<37 years	748	(72)	220	(72)	722	(78)	1690	(75)
≥37 years	291	(28)	84	(28)	203	(22)	578	(25)
**Gender**								
Female	295	(28)	155	(51)	324	(35)	774	(34)
Male	743	(72)	149	(49)	601	(65)	1493	(66)
Transgender	1	(0)	0	(0)	0	(0)	1	(0)
**Race**								
Black, Non-Hispanic	259	(25)	203	(67)	639	(69)	1101	(49)
White, Non-Hispanic	626	(60)	47	(15)	206	(22)	879	(39)
A/PI/AI/AN/Other	98	(10)	6	(2)	38	(4)	142	(6)
Unknown	56	(5)	48	(16)	42	(5)	146	(6)
**Ethnicity**								
Hispanic	54	(5)	43	(14)	42	(5)	139	(6)
Non-Hispanic	985	(95)	261	(86)	883	(95)	2129	(94)
**Risk Behaviors** a								
IDU	18	(2)	11	(4)	10	(1)	39	(2)
Sex partner of IDU	41	(4)	15	(5)	17	(2)	73	(3)
Sex while using non-injection drugs	276	(27)	233	(77)	267	(29)	776	(34)
MSM or sex partner of MSM	304	(29)	16	(5)	47	(5)	367	(16)
Sex partner of person with HIV/AIDS	56	(5)	13	(4)	14	(2)	83	(4)
STD diagnosis	125	(12)	82	(27)	285	(31)	492	(22)

A/PI/AI/AN/Other, Asian/Pacific Islander/American Indian/Alaska Native/Other; IDU, injection drug user; MSM, men who have sex with men; STD, sexually transmitted diseases.

a Risk behaviors are not mutually exclusive.

#### Test results

Of the 2268 clients tested, results of one (0.04%) test using whole blood and six (0.3%) using oral fluid were invalid. This difference in the number of invalid results by specimen type was not statistically significant (Fisher's exact test, *P* = 0.12). Nine (0.4%) clients had reactive tests on both oral fluid and whole blood, and 2253 (99.3%) had non-reactive tests on both specimens (Table 3). Valid oral-fluid and whole-blood test results were 100% concordant, and all reactive rapid tests were confirmed as positive by serum Western blot (100% specificity).

**Table 3:** OraQuick Advance rapid HIV antibody test results for 2268 clients tested with oral fluid, Incidence Study, February 2005 to June 2005.

	Minnesota		New Jersey		Wisconsin		Total	
Test Results	n = 1039	(%)	n = 304	(%)	n = 925	(%)	n = 2268	(%)
**Non-reactive**	1033	(99.4)	302	(99.3)	918	(99.3)	2253	(99.3)
‘T’ zone description:								
No line	1032		302		916		2250	
Visible line appearing gray	0		0		2		2	
Don't know if line visible	1		0		0		1	
**Reactive**	4	(0.4)	2	(0.7)	3	(0.3)	9	(0.4)
Test line intensity^a^:								
Darker than control	1		2		1		4	
As dark as control	2		0		2		4	
Fainter than control	1		0		0		1	
**Invalid**	2	(0.2)	0	(0)	4	(0.4)	6	(0.3)

a Test line intensity of test device compared to test line intensity of low-positive external control.

#### Device characteristics

No participant had an invalid test on both whole-blood and oral-fluid specimens. Of the six invalid oral-fluid results, one had a partial reddish-purple test line, three had a red background that did not clear at 20 minutes, and two developed no control lines. All six clients with invalid oral-fluid results had non-reactive whole-blood results and non-reactive repeat oral-fluid results. Two oral fluid tests classified as non-reactive were described by the same reader as having a faint gray test line (Table 3); both clients had non-reactive finger-stick results. The test devices were used on two different days, were read at 20 and 21 minutes respectively, and were from two different lots. The first client whose oral-fluid test device had a gray line was a 62-year old White male who reported multiple sex partners and tested anonymously. The second client was a 47-year old Asian male who reported sex with a known HIV-infected male partner six weeks prior to the current HIV test; he had a negative HIV test result three months prior to the current test. No further HIV test results are available for either client as they did not return to the study site for subsequent testing.

## Discussion

As part of a two year rapid HIV test performance study, a cluster of 16 false-positive oral fluid OraQuick tests was reported from April 15, 2004 through August 31, 2004 during which the specificity of the test fell below the FDA required minimum of 98% [4], [5]. However, in the first 18 months of the performance study, and in three other contemporaneous studies, oral-fluid OraQuick tests demonstrated a specificity ≥99.6% [4]. The overall specificity of the oral-fluid test for the entire duration of the performance study was 99.0% (95% CI: 98.6–99.4) and there were 23 oral-fluid false positives, 1 oral-fluid false negative, and 2 whole blood false positives [4]. Our field investigation did not suggest a specific cause for the cluster, and our subsequent incidence study detected no false-positive tests. The lack of any false-positive tests despite testing 2268 clients at nine sites during the 4-month incidence study suggests that the observed cluster was isolated and that, even as false-positive results occur at expected rates (we expected two to nine false-positives in the incidence study), future occurrences of false-positive tests may not be evenly distributed over time. While false-positive oral fluid results reported in the performance study occurred in outreach sites, such results have also been reported in a static clinical site (Shelley Facente, personal communication) [7].

The retrospective nature of the field investigation imposed certain limitations. First, we did not observe test operators performing and interpreting the tests at the time they identified the false-positive results. Although we did not note these practices when the operators performed the tests under our observation, over-collection of oral-fluid specimens (swabbing the outer gums more than once), over-interpretation of the test line, or other practices such as specimen contamination and erroneous recording of results might have contributed to false-positive results. The manufacturer intends to systematically evaluate the influence of oral fluid over-collection on test specificity (Stephen R. Lee, OraSure Technologies, personal communication). Second, we were unable to accurately capture the appearance of the false-positive devices at the time they were identified. We used optical densitometry and detected a peak in the test zone of false-positive devices when compared to a non-reactive device but these readings were obtained days to weeks after the test was run and the devices may have changed appearance in the interim. We were therefore unable to make valid comparisons between interpretations of false-positive devices among operators who had identified them versus operators who had not. Third, we were unable to obtain data retrospectively from persons with false-positive results about medical conditions (e.g., Epstein-Barr virus infection, hepatitis A and B infection, rheumatoid factor, or multiparity) which might be associated with false-positive OraQuick Advance results [1].

Older age was significantly associated with false-positive results in the performance study, but age may be a marker for unmeasured conditions that increase with age and that are associated with false-positive results (e.g., prevalence of markers of hepatitis B infection among injection drug users and men who have sex with men). Since no oral-fluid false positives were detected in the incidence study, we were unable to compare age differences between false positives in the performance and incidence study populations. Despite the lack of statistical significance, false-positive results in the performance study occurred more often in specific groups such as injection drug users and men who have sex with men. These findings suggest one or more unmeasured host and site-specific factors may have interacted to produce the cluster. For example, higher rates of viral hepatitis are known to occur in these groups. Both the incidence study and the performance study had similar representation from these groups.

As with any diagnostic test, comprehensive instructions for performing and interpreting the OraQuick test are essential. The OraQuick Advance package insert does not specify how to interpret partial test lines or visible test lines that do not have a discernable reddish-purple color [1]. In the incidence study, two test lines described by the same operator as very faint or gray were interpreted as non-reactive, and one incomplete reddish-purple test line was considered invalid. All three of these test lines occurred among clients with a negative finger-stick rapid test result; since OraQuick is more sensitive with finger-stick whole blood than with oral fluid it is unlikely these were true positives [1], [4]. In the original Minnesota performance study and perhaps elsewhere [7], these types of test results may have been reported as reactive. Without guidance from the manufacturer other clusters of false-positive results might occur, in part because of differential interpretation. The manufacturer should revise the package insert to specify how to interpret partial lines and visible lines in the test zone that do not appear reddish-purple.

With proper training to ensure correct use and interpretation, the OraQuick Advance oral-fluid rapid HIV test can be used successfully as a point-of-care test, although the test has a lower specificity with oral fluid than with whole blood [1], [4], [7]. Regardless of the specimen type, clients should be counseled that a reactive rapid HIV test result is preliminary and must be confirmed [8], [9].

## Footnotes

1 In this paper we refer to both OraQuick and OraQuick Advance as OraQuick.
